# Macroglossia in Beckwith-Wiedemann Syndrome Is Attributed to Skeletal Muscle Hyperplasia

**DOI:** 10.1155/2020/8871961

**Published:** 2020-11-01

**Authors:** Yuzo Oyama, Haruto Nishida, Osamu Kobayashi, Kenji Kawano, Kenji Ihara, Tsutomu Daa

**Affiliations:** ^1^Department of Diagnostic Pathology, Faculty of Medicine, Oita University, Yufu, Japan; ^2^Department of Pediatrics, Faculty of Medicine, Oita University, Yufu, Japan; ^3^Department of Oral and Maxillofacial Surgery, Faculty of Medicine, Oita University, Yufu, Japan

## Abstract

Macroglossia is a common feature in patients with Beckwith-Wiedemann syndrome (BWS). The underlying cause of macroglossia in BWS remains unknown, and further histological studies are required to uncover its etiology. We present the case of a 5-year-old girl who was diagnosed with BWS and underwent partial tongue resection for difficulties in articulation and aesthetics. The keyhole-shaped partial resection revealed a harder posterior side than the anterior. Microscopically, the posterior side consisted of dense subepithelial eosinophilic areas composed of an abundance of tightly packed skeletal muscle fibers that were arranged in a fascicular or storiform pattern. BWS-associated macroglossia results from skeletal muscle hyperplasia, consistent with true macroglossia. Therefore, tongue resection may be beneficial for such patients. Further studies are required to develop personalized surgical interventions for each patient with BWS.

## 1. Introduction

Beckwith-Wiedemann syndrome (BWS) is an overgrowth disorder in children caused by epigenetic changes on chromosome 11p15. Imprinted genes present in this locus include insulin-like growth factor 2 (*IGF2*/*H19*) and cyclin-dependent kinase 1C (*CDKN1C*/p57^KIP2^). Hypermethylation of IGF2/H19, hypomethylation of *CDKN1C*/p57^KIP2^, or paternal uniparental disomy (pUPD) is commonly associated with BWS. BWS is characterized by macroglossia, macrosomia, abdominal wall defects, ear creases, facial nevus, flammeus, renal abnormalities, visceromegaly, neonatal hypoglycemia, hemihyperplasia, and increased risk of embryonal tumors [1].

Macroglossia leads to obstructed airways, difficulties with articulation, mandibular protrusion, and uneven teeth spacing that results in obstructive sleep apnea in children with BWS [2]. Macroglossia can be caused by multiple factors, such as tumors, systemic disorders, vascular malformations, muscular and functional disabilities, local reactive changes, and drugs. However, little is known about the etiology and histology of macroglossia in patients with BWS [3–5]. Here, we report a case of a child with BWS and performed a histological assessment of the associated macroglossia.

## 2. Case Presentation

**Figure 1:**
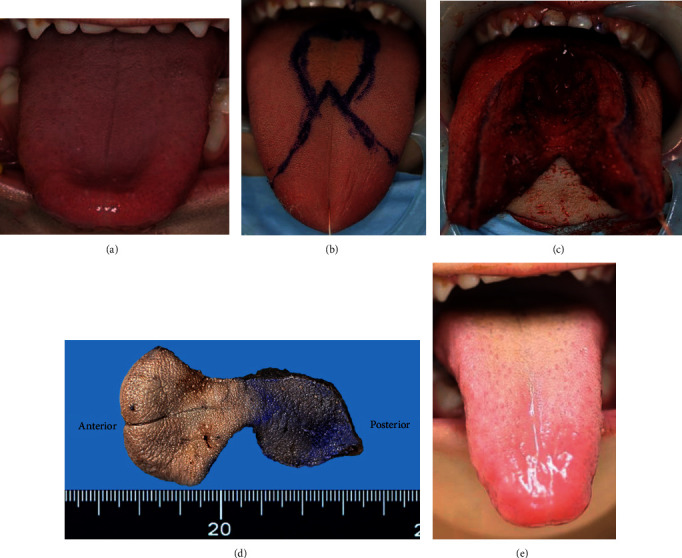
Gross appearance. The preoperative tongue (a), the operation design (b), the intraoperative status (c), the resected tongue (d), and the postoperative tongue (e).

A 5-year-old girl was referred to Oita University Hospital for partial reduction of macroglossia. She was born premature and required urgent neonatal care for respiratory and swallowing difficulties that were caused by macroglossia. She was diagnosed with BWS based on overgrowth, macroglossia, exomphalos, bilateral linear earlobe creases, and nevus flammeus on the forehead between the eyebrows. She did not exhibit neonatal hypoglycemia, visceral hyperplasia, or embryonal tumors. However, she displayed mild intellectual disability. Reductive surgery for macroglossia was recommended during infancy. However, the surgery was delayed until she was older out of concern over general anesthesia. There were no changes in malocclusion or mandibular prognathism for over 5 years. She did not complain of oral discomfort and underwent surgery to prevent problems with articulation (Figure 1(a)). The tongue was partially resected using the keyhole-shaped technique (Figures 1(b)–1(d)). There were no postoperative complications or recurrence of macroglossia for 2 years (Figure 1(e)).

**Figure 2:**
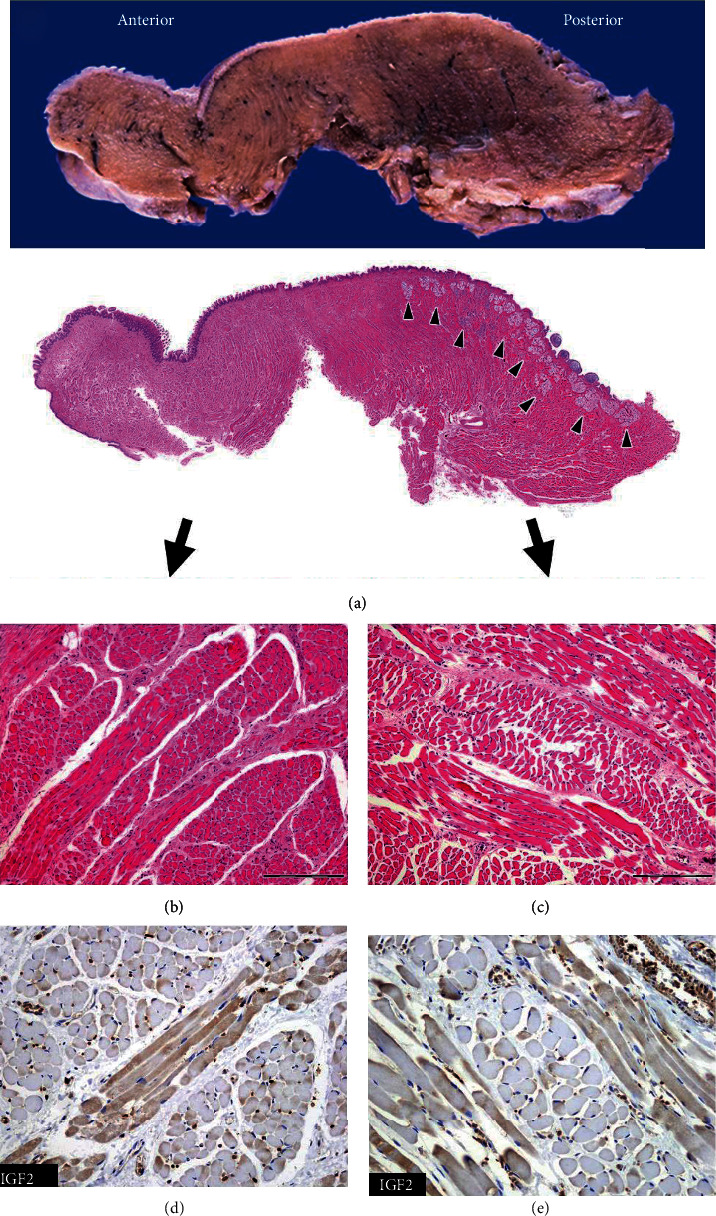
The autopsy tongue specimen of a 1-year and 8-month-old girl (control). The sagittal section has been shown. The subepithelial tissues in the posterior side are composed of muscle fibers and minor salivary glands (arrowhead) (a) (loupe). Muscle fibers are composed of longitudinal and cross section regularly, and interstitial spaces between muscle fibers can be seen on both sides (b and c) (100x, scale bar: 200 *μ*m). IGF2 protein expression in the muscle fibers does not differ between the anterior (d) (200x) and posterior sides (e) (200x).

**Figure 3:**
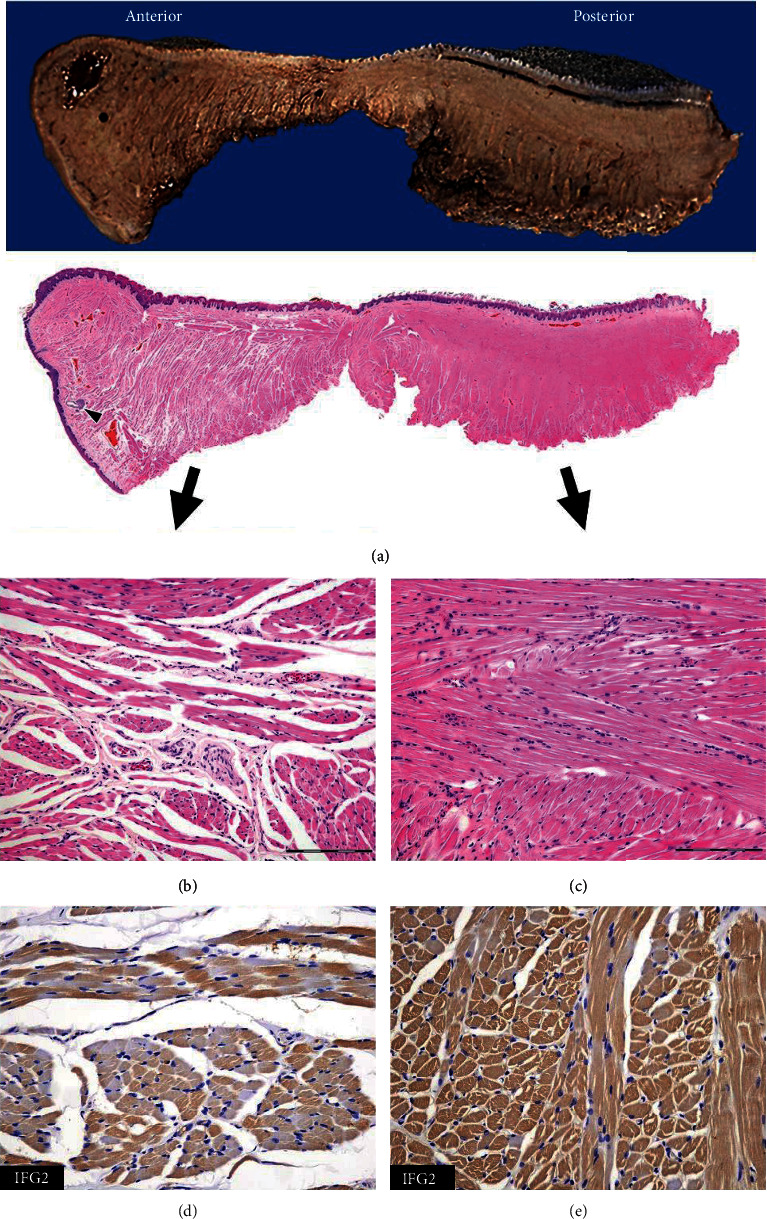
Histology of macroglossia associated with Beckwith-Wiedemann syndrome in the present case. The sagittal section shows an enlarged subepithelial eosinophilic area that is more prominent on the posterior side. Few salivary glands can be seen (arrowhead) (a) (loupe). On the anterior side, loose interstitial spaces are present between the skeletal muscle bundles (b) (100x, scale bar: 200 *μ*m). On the posterior side, several skeletal muscle bundles are closely arranged in a storiform or fascicular pattern (c) (100x, scale bar: 200 *μ*m). Variable and heterogeneous IGF2 protein levels are seen on the anterior side (d) (200x) compared to the more intense and homogenous staining on the posterior side (e) (200x).

The resected keyhole-shaped tongue was ~5 cm in size. There was no evidence of a tumor on the outer surface. As a control, we examined the autopsied tongue of a 1-year and 8-month-old girl who did not have a growth disorder (Figures 2 and 3). In the patient, a sagittal section of the tongue revealed overall hardness and thickening. The posterior side (body of the tongue) was harder than the anterior (the anterior of the tongue; Figure 3(a)).

At low magnification, the hematoxylin-and-eosin-stained patient specimen exhibited a dense subepithelial eosinophilic area in the posterior side (Figures 2(a) and 3(a)). Subepithelial minor salivary glands were scarce in the patient tongue but easily detectable in the control (Figures 2(a) and 3(a)). No hyperplasia or hypertrophy of the epithelium was detected. Loose interstitial spaces were observed on both sides of the control specimen, but only on the anterior side of the patient specimen (Figures 2(b), 2(c), and 3(b)). The dense subepithelial eosinophilic area consisted of longitudinal sections of skeletal muscle bundles that were prominent on the posterior side and tightly packed in a storiform or fascicular pattern (Figure 3(c)). The average diameters of the muscle fibers per cross section in the anterior and posterior sides were 18 *μ*m and 22 *μ*m in the control and 18 *μ*m and 24 *μ*m in the patient, respectively. The maximum number of longitudinal sections at 100x magnification was 20 and 33 fibers on the posterior and the anterior sides, respectively, of the patient specimen and 28 fibers on either side of the control. In the control specimen, IGF2 protein was more highly expressed in the longitudinal sections compared with the transverse sections, and the staining pattern or intensity did not differ between the anterior and the posterior sides of the tongue (Figures 2(d) and 2(e)). However, in the patient specimen, IGF2 protein was detected to similar extent in both sections, and more intense staining was observed on the posterior side compared with the anterior (Figures 3(d) and 3(e)).

## 3. Discussion

Three reports describe the histology of macroglossia in patients with BWS (Table 1) [3–5]. Arons et al. investigated two patients with BWS and suggested that the increase in tongue size might reflect skeletal muscle hyperplasia because neither case displayed muscle fiber hypertrophy, fat accumulation, or fibrosis [3]. Additionally, Hamazaki and Saito observed mild muscle fiber hypertrophy and associated fibrous degeneration in the tongue of an autopsied patient with BWS [4]. Recently, Yamada et al. showed that a pUPD-associated BWS case exhibited hemihyperplasia on the right side of the partially resected tongue, which had a higher number of closely spaced skeletal muscle fibers than the left side [5]. In the present study, the posterior side of the patient tongue had fewer minor salivary glands and less loose connective spaces than the control but had abundant longitudinal muscle fibers. Our histological findings were similar to those of Yamada et al., except for the location of the skeletal muscle hyperplasia. The average diameter of muscle fibers showed no marked difference between the tongues of the control and the patient, irrespective of location; however, there were marked differences in the number of muscle fibers between the two. In the patient, the posterior side of the tongue had a larger number of fibers compared with the anterior side, in contrast to the control.

Consequently, we consider “hyperplasia” a more appropriate term than “hypertrophy” for describing macroglossia in BWS because the skeletal muscles in the tongue increased in number rather than in size. These findings indicate the presence of true macroglossia in BWS caused by skeletal muscle hyperplasia. The diffuse intense staining of IGF2 in the hyperplastic region of the longitudinal and cross sections explains the increased number of skeletal muscle fibers. IGF2 is a growth factor that is essential for skeletal muscle growth. Therefore, its upregulated expression leads to an increase in skeletal muscle mass [6]. The IGF2 staining in the longitudinal muscle fiber sections of the control specimen indicated that IGF2 is primarily located in the cytomembrane. Moreover, although our report did not include genetic analysis, epigenetic alterations such as hypermethylation at the *IGF2* locus or pUPD may be implicated.

The treatment of macroglossia depends on its type. For example, Down syndrome is associated with relative macroglossia and surgical procedures such as mandibular distraction or oral cavity widening, which improve the symptoms more effectively than reducing the tongue volume [7]. However, for BWS patients with macroglossia, tongue reduction surgery remains the mainstay of treatment. It is reasonable to decrease the tongue volume in true macroglossia because tongue resection can correct the cause and problems associated with open bite, aesthetics, malocclusion, and articulation. Patients with BWS exhibit different locations and degrees of skeletal muscle hyperplasia. Preoperative and intraoperative assessment and careful palpation will confirm hardening or asymmetry, as well as facilitate the demarcation of the afflicted regions of the tongue. It is important to note that tongue resection may not correct the problems associated with airway obstruction in BWS, because tonsillar and adenoidal hypertrophy may be the underlying cause [8].

Furthermore, the optimal time for surgical intervention is yet to be determined. There is a risk of tongue regrowth or orofacial deformity associated with premature or delayed tongue reduction, respectively [9]. Kopriva and Classen proposed that surgery should be delayed until 6 months of age, owing to the reduction in the rate of tongue growth [10]. Considering jaw deformity and dental occlusion, Yamada et al. recommend performing glossectomy in patients up to 1.6 years of age [5]. In our case, the patient underwent tongue resection at a relatively late age as a result of concerns about the growth of the child. Further studies are required to determine the optimal period for the surgical treatment of macroglossia associated with BWS.

In summary, we have presented a histological account of macroglossia in a girl with BWS compared with a control. We found that skeletal muscle hyperplasia resulted in true macroglossia in the patient. Tongue reduction for true macroglossia may be effective depending on the individual case. The development of guidelines for personalized surgical treatment of patients with BWS is required.

## Figures and Tables

**Table 1:** Previous reports on macroglossia in patients with Beckwith-Wiedemann syndrome.

Reference	Year	Age (months)	Sex	Muscle	Fat	Fibrosis	Observation
Arons et al. [3]	1970	39	Female	Hypertrophy	No	No	The increase in tongue size may reflect hyperplasia of the muscle
Arons et al. [3]	1970	65	Male	Hypertrophy	No	No	The increase in tongue size may reflect hyperplasia of the muscle
Hamazaki and Saito [4]	1979	3	Male	Mild hypertrophy	No	Yes	
Yamada et al. [5]	2018	12	Female	Hyperplastic	No	No	The hemihyperplastic side contains closely spaced normal sized muscle fibers
Our case	2018	67	Female	Hyperplastic	No	No	The posterior muscle fibers exhibit fascicular or storiform pattern

## Data Availability

No data were used to support this study.
